# Supplementary material for: Efficacy of Large Use of Combined Hypofractionated Radiotherapy in a Cohort of Anti-PD-1 Monotherapy-Treated Melanoma Patients
Source: Cancers (Basel). 2022 Aug 23;14(17):4069. doi: 10.3390/cancers14174069 (PMC9454723; doi:10.3390/cancers14174069)
Supplement: Supplementary file 1 [file cancers-14-04069-s001.zip › File S1.pdf]

Protocol

Retrospective evaluation of HYPO-fractionated radiotherapy in anti-PD-1 treated MELanoma patients (R-HYPO-MEL)

Ph SAIAG
V3 March 5 2021

INTRODUCTION

Immune checkpoint inhibitors (ICI) have improved the prognosis of advanced melanoma patients(1), particularly the anti-programmed death-1 (PD-1) monoclonal antibodies (mAb) nivolumab and pembrolizumab. First line nivolumab or pembrolizumab has led to better progression-free survival (PFS) and overall (OS) or melanoma specific survival (MSS) when compared to chemotherapy or ipilimumab, an anti-CTLA-4 mAb (2, 3). Depending on the line of treatment, anti-PD-1 monotherapy in advanced melanoma patients without brain metastasis is associated with complete and partial response (CR and PR) rates ranging from 20 to 40% and a median PFS of 4.1 to 6.9 months (1). Furthermore, brain metastases, which convey a poor prognosis, have been reported for as many as 40% of metastatic melanoma patients (4-7), and account for 10 to 20% of all patients with cerebral metastases (8). A recent systematic review of 122 randomized controlled trials of metastatic cutaneous melanoma treatments revealed that all but 29 of these studies excluded patients with melanoma brain metastases (MBM) (9).

Thus, better strategies accounting for this patient population are urgently required. Combined nivolumab with ipilimumab was demonstrated to provide higher response rates in melanoma patients without brain metastasis, but the demonstration of longer PFS and MSS when compared to nivolumab alone is lacking and severe adverse events (SAE) are over twice as frequent among patients that received the combined therapies (10). This combination was also demonstrated to provide in two non-randomized studies high response rates, and encouraging PFS data in melanoma patients with 1-4 asymptomatic brain metastases (11, 12), again at the cost of high toxicity.

Radiotherapy exerts a whole range of actions upon the immune system (13), and preclinical data suggest that ICI combined with radiotherapy could improve tumor response (14). A systematic review of radiotherapy combined with ipilimumab in melanoma patients revealed a 26.5% abscopal response rate (i.e. the regression or disappearance of tumor(s) outside of the radiation field(s))(15). Although some studies have reported on the effect of combining non-brain directed radiotherapy and anti-PD-1 mAb, its role in melanoma care is still debated due to the frequent use of retrospective designs, inclusion of small numbers of patients, and either multisite radiotherapy (16) or sub-optimal radiation scheduling (i.e., radiotherapy before anti-PD-1, insufficient dosing/session)(16, 17). In most studies, no control group was present (18). We have previously reported that anti-PD-1 mAb and concurrent hypo-fractionated radiotherapy induced a CR+PR rate of 36-38% that lasted for a long period in advanced melanoma patients who either received early emergency radiotherapy (19) because of life-threatening locations, or who had previously failed anti-PD-1 monotherapy and received hypo-fractionated radiotherapy combined with an unmodified anti-PD-1 mAb regimen (20).

Hypo-fractionated radiotherapy delivers higher doses than standard palliative radiotherapy during a reduced number of sessions and may be more active when combined with an ICI.(21) The above-mentioned results support the hypothesis that hypo-fractionated radiotherapy could be the optimal extracranial radiotherapy to use in melanoma patients in combination with an anti-PD-1 mAb. In cell culture and animal models, radiation-induced immunogenic cell death occurs through the release of damage-associated molecular patterns (DAMP), such as double-stranded DNA, which activates secretion of type-1 interferons (IFN) through the GMP-AMP synthase/stimulator of IFN genes (cGAS/STING) pathway.(14) DNA exonuclease 3' repair exonuclease 1 (Trex1) was induced by radiation doses above 12-18 Gy in different cancer cells and attenuated their immunogenicity by degrading DNA.(22) Fractionated protocols of radiotherapy were more effective in inducing immune-mediated abscopal effects than a single ablative dose and may overcome

radiotherapy-induced adaptive resistance by upregulation of PD-L1.(23) Repeated radiations at doses (8Gy x 3) just above the threshold of Trex1 induction greatly amplified type-1 IFN production, resulting in recruitment and activation of Batf3-dependent dendritic cells which are essential for priming of CD8+ T cells that mediate systemic tumor rejection in the context of ICI.(22) In preclinical studies, the optimal dosing and fractionation strategy for each cancer type has not yet been determined, but larger doses per fraction were associated with enhanced abscopal effects.(23) In humans, high fraction doses were the source of most reported abscopal effects observed without concurrent ICI.(15) In humans, a trial of ipilimumab combined with hypo-fractionated radiotherapy (6GyX5 or 9GyX3) in 39 patients with non-small cell lung cancer (where ipilimumab had failed to demonstrate significant efficacy alone or in combination with chemotherapy), provided a proof-of-concept, with radiological responses observed in 18% of patients along with type-1 IFN release.(21)

Few studies have investigated the association of ICI with concurrent brain radiotherapy, which is currently mainly given as stereotactic radiosurgery (SRS), in melanoma brain metastases (24). SRS delivers very high doses per fraction, far above the standard 3 Gy in 10 fractions used standard *in toto* brain radiotherapy. However, logistical constraints for brain radiotherapy and, when a gamma-knife device is used, the need to screw under anesthesia an helmet to the scalp bones, prevent repeated dosing. In a retrospective study with a median follow-up of 15 months, the 6-month and 12-month intracranial PFS rates were 69% (95%CI,54-87%) and 42% (95%CI,24-65%) for the 35 patients receiving SRS and nivolumab. The 12-month extracranial PFS and OS were 37% and 78%, respectively (25). In 25 patients with brain metastasis treated with pembrolizumab and SRS followed for a median time of 8.4 months, local control was achieved in 68% of patients, and the median OS was 15.3 months (95%CI,4.6-26 months) (26).

Our objectives are to evaluate hypo-fractionated radiotherapy and/or SRS combined with anti-PD-1 mAb continuation in a large cohort of consecutive advanced melanoma patients treated with anti-PD-1 mAb monotherapy. To date, in our skin cancer specialized department, more than 200 advanced unresectable melanoma patients have received anti-PD-1 monotherapy and because of our previous works, we have largely prescribed hypo-fractionated radiotherapy. We have entered patients characteristics and have collected prospectively the result of radiological evaluations in the clinical system of our hospital (ORBIS).

The aims of this retrospective study are:

1/ to look if large use of hypo-fractionated radiotherapy or SRS in routine care can increase the rate of complete response. Complete responses have been observed in less than 15-19% of included patients in registration trials. Our policy of frequent use of radiotherapy in case of progression while on anti-PD-1 monotherapy or in case of emergency in the same situation may have raised the % of complete response to ≥30%. Achieving CR with anti-PD-1 mAb seems an important goal to achieve, as such patients experience long lasting CRs even when the anti-PD-1 administration has bee stopped.

2/ to compare Kaplan Meier curves of MSS and PFS in radiated and non-radiated patients, taking as origin anti-PD-1 initiation and, for patients with radiotherapy, comparing those responding and those not responding from the date of first radiotherapy session.

3/ to assess the rate of patients with CR who can stop treatment without relapse.

4/ To better characterize the profile of patients achieving PR or CR with hypo-fractionated radiotherapy by comparing those achieving PR + CR to those achieving S and P by studying known prognostics factors of advanced melanoma or of radiotherapy: elevated LDH serum level, ECOG performance status, N of metastatic sites, AJCC8th edition staging, presence of brain of liver metastasis, N previous treatments of advanced melanoma,

previous use of BRAF+MEK inhibitors in BRAFV600-mutant melanoma, presence of oligometastatic disease[1], presence of oligoprogression[2] (27), indication for radiotherapy (emergency early radiotherapy versus late radiotherapy for confirmed progression), number of radiotherapy sessions, organs radiated (lymph node/no lymph node), mutational status. Rather than an intra-cohort case-control study, a propensy score will be used if predicitve factors are suspected.

4/ to precise on a large number of radiated patients the percentage of patients with abscopal effect

5/ to precise the kinetics of response in responding patients (after which delay first response is seen?)

6/ to compare SRS, hypo-fractionated radiotherapy, and conventional radiotherapy

7/ to assess the percentage of patients with CR and treatment cessation, and the probability of recurrence, and effect of retreatment.

Patients and methods

Most data are prospectively collected in our skin cancer department since January 1, 2014 (date of early access programs and commercialization of anti-PD-1 mAb) and

[1] A type of metastasis in which cancer cells from the original (primary) tumor travel through the body and form a small number of new tumors (metastatic tumors) in one or two other parts of the body

[2] **Oligoprogression** is an increasingly recognized concept in oncology, denoting a state where after an initially successfully systemic therapy of disseminated metastases, a single or very few lesions display further progression. A key difference between the related concepts of oligometastasis and oligoprogression is that a patient with the latter can have generally any number of metastases as long as only a solitary or a select few show progression, with the rest displaying either regression or stability while the patient continues to receive systemic antitumor therapy [1]. By definition, the state of oligoprogression occurs after initially successful therapy of polymetastatic disease, where disease progression is encountered only in a minority of the affected sites [2]. The criteria of oligoprogression have not yet solidified, with some studies describing a precise number e.g. four or fewer sites of progression in NSCLC as the prerequisite for it

maintained to date for melanoma patients treated with anti-PD-1 mAb and not included in industry-sponsored clinical trials. *BRAF*^{V600} and *NRAS*^{Q61} mutational status are assessed as previously reported.(28, 29) Nivolumab was given intravenously initially every 2 weeks at a dose of 3 mg/kg and pembrolizumab at 2 mg/kg every 3 weeks, according to product labels, until unambiguous PD, unacceptable side effects, or clinician decision to discontinue treatment. Fixed dose schedules were also used when this new regimen granted marketing authorization in France. Additionally, anti-PD-1 mAb was continued beyond progression at first evaluation to allow for pseudo-progression(30) or later if at least one lesion could be treated with a local treatment such as radiotherapy or surgery.

Patients are followed according to our standard procedures, which require a medical consultation with standardized questionnaire before each infusion and standardized blood tests before and at every other infusion of anti-PD-1 mAb. Efficacy was evaluated every 3 months using thoracic, abdominal and pelvic computed tomography (CT) scans, head CT scan or magnetic resonance imaging, carried out by radiologists experienced in melanoma. In addition, normal (18)F-labeled fluorodeoxyglucose-positron emission tomography (FDG-PET) scans were required to confirm CR or to address ambiguous CT images. All images were analyzed on a weekly basis during a joint meeting with radiologists, with measuring of target lesions according to Response Evaluation Criteria In Solid Tumors version 1.1 (RECIST 1.1) guidelines(31) and evaluation of tumor response. All tumor evaluations were carried out blinded to characteristics of radiotherapy (except the site) and stored prospectively in the patient files. Adverse events were routinely graded according to the Common Terminology Criteria for Adverse Events (CTCAE) version 5.0.

Our specialized melanoma tumor board provided the indications for concomitant radiotherapy which could be performed either within the first 3 months of PD-1 blockade for rapidly progressing symptomatic or life-threatening lesion(s), or later in patients with PD or in patients with long-lasting SD on anti-PD-1 therapy. The radiotherapy regimen was standardized for most patients: for extra-cranial lesions, patients received 3-5 doses; for

cranial radiotherapy, patients received stereotactic radiosurgery (SRS) in one or two sessions delivered through a Gamma-knife or another stereotactic procedure.

For this study, the collection of data will be locked on April 30, 2021, and we will search for records of <u>all</u> patients with confirmed inoperable, AJCC stage IIIC-IV cutaneous or mucous membrane melanoma treated with pembrolizumab or nivolumab monotherapy, regardless of *BRAF* and *NRAS* mutational status and number and type of previous therapies, between January 1, 20154, and August 30, 2019.

Key exclusion criteria will be: age <18 years, association with ipilimumab, ECOG status >2, inclusion in a randomized industry-sponsored trial for which we were blinded to the treatment received.

We will search for the patients who received concomitant radiotherapy (defined as at least one dose given during pembrolizumab or nivolumab treatment and one month after last dose). All radiotherapy procedures performed during study window will be recorded. Conventional radiotherapy will be defined around 2 Gy/fraction, with 5 fractions/week. Hypofractionated radiotherapy will be defined as >4 Gy/fraction and less fractions. Stereotactic radiosurgery (SRS) will be defined use of ablative single doses between 9 and 24 Gy and one or very few fractions, with very precise irradiation of small tumor volumes.

Patients will then be divided in 2 groups: those with concomitant radiotherapy and those without radiotherapy. Those with concomitant radiotherapy will be divided in 2 subgroups: those with rapidly progressing symptomatic lesions or threatening location(s) receiving radiotherapy within the first 3 months of PD-1 blockade ("emergency radiotherapy group"); and those with PD either slowly or after first response or long-lasting SD (confirmed on two consecutive CT-scans) on anti-PD-1 therapy ("late radiotherapy group").

Data extraction

The following information will be extracted from the ORBIS clinical database and retrospectively analyzed:

For all patients, treatment initiation date and final date of anti-PD-1 mAb, age at anti-PD-1 initiation, gender, primary melanoma characteristics (date of diagnosis of primary and of metastatic disease, histologic subtype of primary, Breslow, ulceration, mitotic index, *BRAF, NRAS and cKit* mutational status), previous systemic therapies for advanced melanoma, and, at initiation of anti-PD-1 mAb, AJCC8th edition staging, Eastern Cooperative Oncology Group (ECOG) performance status, metastatic site(s), lactate dehydrogenase (LDH) serum levels (<UNL, >1UNL<2, >2UNL), presence of brain or liver metastasis, dates and results of all radiological and final survival assessments, treatments given after progression if any.

For patients with concomitant radiotherapy, we will add dates and characteristics of the radiotherapy sessions (date of first fraction, N fraction, N Gy/fraction, total dose, targets), its intention (early emergency/late radiotherapy), some further characteristics of the melanoma (oligometastatic, oligoprogression), responses in radiated and non-radiated lesions (and thus the presence of abscopal effect), and toxicity.

Endpoints

The primary endpoints will be responses according to RECIST 1.1 criteria. The baseline images for evaluating radiotherapy and PD-1 blockade will be those taken immediately prior to radiotherapy. According to the RECIST 1.1 criteria,(31) CR will be defined as the disappearance of all lesions (with lymph nodes having reached a dimension <10 mm in their smallest axis), PR as a decrease by at least 30% of the sum of the diameters of the target lesions, PD as an increase >20% of the sum of the diameters of the target lesions or occurrence of any new lesions, and SD as having neither sufficient shrinkage to qualify for PR nor sufficient increase to qualify for PD. For radiated patients, separate evaluation will be performed as previously published (19) in radiated and non-radiated areas using RECIST 1.1 criteria, with abscopal effect defined as PR or CR outside radiated fields.

Secondary endpoints will be MSS (time from the first dose of anti-PD1 mAb to death from melanoma), PFS (time from first dose to documented PD or death), and safety. We will also calculate MSS and PFS from the first day of radiotherapy.

Ethics

According to French Law, this study abides by standard medical practices and does not require a written informed consent. However, consent was obtained orally from all patients. In addition, numerous patients gave written informed consent to participate in one or two national prospective cohorts of advanced melanoma (MelBase: NTC028228202, RIC-Mel: NCT03315468). Study will be conducted according to the principles of the declaration of Helsinki.(32)

Methods of informing the population concerned

All patients will be fully and fairly informed, in understandable terms, of the objectives of the study, of their rights to refuse to participate in the study or of the possibility to withdraw from the study at any time. Individual information concerning research activities in the institution must be provided to the persons concerned.

If the patient objects to the use of his or her data, a member of the medical team must mention this in his or her medical file.

Steps relating to IT regulations and freedom

1.1.1. Data processing in France

Commitment to comply with the "Reference Methodology" MR 004

This research falls within the framework of the "Reference Methodology for the processing of personal data implemented in the framework of research in the field of health" (MR-004 modified). AP-HP, the promoter of the research, has signed an undertaking to comply with this "Reference Methodology".

The law stipulates that the declaration of the computerised file of personal data collected for the research must be made before the actual start of the research.

Statistics

If difference are observed in radiated and non-radiated patients, a propensity score matching (PSM) will be performed to match treatment groups (with/without radiotherapy) according to patient characteristics. For PSM, all covariates that could have an influence on outcome will be included (age, ECOG PS status, brain metastases, previous therapy, AJCC8th edition staging, LDH serum level, N of sites with metastasis ≥3, <3). We will measure covariate balance by calculating the z-difference [63]. Statistical analyses will be performed separately for the entire patient cohort and for the PSM cohort. All statistical analyses will be performed using SPSS v. 24 (IBM Corp, Armonk, NY, USA) and R v. 3.4.3 (R Core Team, Vienna, Austria: R Foundation for Statistical Computing, 2017). Patient and disease characteristics, as well as treatment-related toxicities, will be compared by the Kruskal–Wallis test for continuous variables and Pearson’s chi-square test for categorical variables where appropriate. PFS and MSS will be estimated by the Kaplan–Meier method and curve comparisons were calculated using the log-rank test. Multivariable Cox proportional hazards regression will be performed to evaluate the effect of multiple covariates simultaneously on outcomes. In any case, p-values < 0.05 will be considered significant and refer to two-sided tests. Quantitative data will be expressed as median and range, qualitative data as frequency and percent.

Number of patients to include: With >200 patients treated in the study period, of whom ≥100 have received concurrent radiotherapy, we have enough power to demonstrate an increase of the CR rate from 15-20% to 30%.

1. Luke JJ, Flaherty KT, Ribas A, Long GV. Targeted agents and immunotherapies: optimizing outcomes in melanoma. Nat Rev Clin Oncol. 2017;18(8):463-82.
2. Ascierto PA, Long GV, Robert C, Brady B, Dutriaux C, Di Giacomo AM, et al. Survival outcomes in patients with previously untreated BRAF wild-type advanced melanoma treated with nivolumab therapy: three-year follow-up of a randomized phase 3 trial. JAMA oncology. 2019;5(2):187-94.
3. Hamid O, Robert C, Daud A, Hodi FS, Hwu WJ, Kefford R, et al. Five-year survival outcomes for patients with advanced melanoma treated with pembrolizumab in KEYNOTE-001. Ann Oncol. 2019;30(4):582-8.
4. Goyal S, Silk AW, Tian S, Mehnert J, Danish S, Ranjan S, et al. Clinical Management of Multiple Melanoma Brain Metastases: A Systematic Review. JAMA Oncol. 2015;1(5):668-76.
5. Davies MA, Liu P, McIntyre S, Kim KB, Papadopoulos N, Hwu WJ, et al. Prognostic factors for survival in melanoma patients with brain metastases. Cancer. 2011;117(8):1687-96.
6. Sampson JH, Carter JH, Jr., Friedman AH, Seigler HF. Demographics, prognosis, and therapy in 702 patients with brain metastases from malignant melanoma. J Neurosurg. 1998;88(1):11-20.
7. Flanigan JC, Jilaveanu LB, Faries M, Sznol M, Ariyan S, Yu JB, et al. Melanoma brain metastases: is it time to reassess the bias? Curr Probl Cancer. 2011;35(4):200-10.
8. Taillibert S, Le Rhun E. [Epidemiology of brain metastases]. Cancer Radiother. 2015;19(1):3-9.
9. Pasquali S, Hadjinicolaou AV, Chiarion Sileni V, Rossi CR, Mocellin S. Systemic treatments for metastatic cutaneous melanoma. Cochrane Database Syst Rev. 2018;2:CD011123.
10. Hodi FS, Chiarion-Sileni V, Gonzalez R, Grob JJ, Rutkowski P, Cowey CL, et al. Nivolumab plus ipilimumab or nivolumab alone versus ipilimumab alone in advanced melanoma (CheckMate 067): 4-year outcomes of a multicentre, randomised, phase 3 trial. Lancet Oncol. 2018;19(11):1480-92.
11. Tawbi HA, Forsyth PA, Algazi A, Hamid O, Hodi FS, Moschos SJ, et al. Combined Nivolumab and Ipilimumab in Melanoma Metastatic to the Brain. N Engl J Med. 2018;379(8):722-30.
12. Long GV, Atkinson V, Lo S, Sandhu S, Guminski AD, Brown MP, et al. Combination nivolumab and ipilimumab or nivolumab alone in melanoma brain metastases: a multicentre randomised phase 2 study. Lancet Oncol. 2018;19:672-81.
13. Barker HE, Paget JT, Khan AA, Harrington KJ. The tumour microenvironment after radiotherapy: mechanisms of resistance and recurrence. Nat Rev Cancer. 2015;15(7):409-25.
14. Rodriguez-Ruiz ME, Vanpouille-Box C, Melero I, Formenti SC, Demaria S. Immunological mechanisms responsible for radiation-induced abscopal effect. Trends Immunol. 2018;39(8):644-55.
15. Chicas-Sett R, Morales-Orue I, Rodriguez-Abreu D, Lara-Jimenez P. Combining radiotherapy and ipilimumab induces clinically relevant radiation-induced abscopal effects in metastatic melanoma patients: A systematic review. Clin Transl Radiat Oncol. 2018;9:5-11.

16. Doyen J, Picard A, Naghavi AO, Thyss A, Passeron T, Lacour JP, et al. Clinical outcomes of metastatic melanoma treated with checkpoint inhibitors and multisite radiotherapy. JAMA Dermatol. 2017;153(10):1056-59.
17. Liniker E, Menzies AM, Kong BY, Cooper A, Ramanujam S, Lo S, et al. Activity and safety of radiotherapy with anti-PD-1 drug therapy in patients with metastatic melanoma. Oncoimmunology. 2016;5(9):e1214788.
18. Sundahl N, Seremet T, Van Dorpe J, Neyns B, Ferdinande L, Meireson A, et al. Phase 2 trial of nivolumab combined with stereotactic body radiotherapy in patients with metastatic or locally advanced inoperable melanoma. International journal of radiation oncology, biology, physics. 2019(104):828-35.
19. Roger A, Finet A, Boru B, Beauchet A, Mazeron JJ, Otzmeguine Y, et al. Efficacy of combined hypo-fractionated radiotherapy and anti-PD-1 monotherapy in difficult-to-treat advanced melanoma patients. Oncoimmunology. 2018;7(7):e1442166.
20. Funck-Brentano E, Baghad B, Fort M, Aouidad I, Roger A, Beauchet A, et al. Efficacy of late concurrent hypofractionated radiotherapy in advanced melanoma patients failing anti-PD-1 monotherapy. Int J Cancer. 2020.
21. Formenti SC, Rudqvist NP, Golden E, Cooper B, Wennerberg E, Lhuillier C, et al. Radiotherapy induces responses of lung cancer to CTLA-4 blockade. Nat Med. 2018;24(12):1845-51.
22. Vanpouille-Box C, Alard A, Aryankalayil MJ, Sarfraz Y, Diamond JM, Schneider RJ, et al. DNA exonuclease Trex1 regulates radiotherapy-induced tumour immunogenicity. Nature communications. 2017;8:15618.
23. Ngwa W, Irabor OC, Schoenfeld JD, Hesser J, Demaria S, Formenti SC. Using immunotherapy to boost the abscopal effect. Nat Rev Cancer. 2018;18(5):313-22.
24. Weaver BD, Goodman JR, Jensen R. Concurrent Radiosurgery and Systemic Therapies for Melanoma Brain Metastases: A Systematic Review. Cureus. 2019;11(11):e6147.
25. Minniti G, Anzellini D, Reverberi C, Cappellini GCA, Marchetti L, Bianciardi F, et al. Stereotactic radiosurgery combined with nivolumab or Ipilimumab for patients with melanoma brain metastases: evaluation of brain control and toxicity. Journal for immunotherapy of cancer. 2019;7(1):102.
26. Nardin C, Mateus C, Texier M, Lanoy E, Hibat-Allah S, Ammari S, et al. Tolerance and outcomes of stereotactic radiosurgery combined with anti-programmed cell death-1 (pembrolizumab) for melanoma brain metastases. Melanoma Res. 2018.
27. Comito F, Leslie I, Boos L, Furness A, Pickering L, Turajlic S, et al. Oligoprogression After Checkpoint Inhibition in Metastatic Melanoma Treated With Locoregional Therapy: A Single-center Retrospective Analysis. Journal of immunotherapy (Hagerstown, Md : 1997). 2020.
28. Moreau S, Saiag P, Aegerter P, Bosset D, Longvert C, Helias-Rodzewicz Z, et al. Prognostic Value of BRAF (V600) Mutations in Melanoma Patients After Resection of Metastatic Lymph Nodes. Ann Surg Oncol. 2012;19(13):4314-21.
29. Helias-Rodzewicz Z, Funck-Brentano E, Terrones N, Beauchet A, Zimmermann U, Marin C, et al. Variation of mutant allele frequency in NRAS Q61 mutated melanomas. BMC dermatology. 2017;17(1):9.
30. Hodi FS, Hwu WJ, Kefford R, Weber JS, Daud A, Hamid O, et al. Evaluation of immune-related response criteria and recist v1.1 in patients with advanced melanoma treated with pembrolizumab. J Clin Oncol. 2016;34(13):1510-7.

31. Eisenhauer EA, Therasse P, Bogaerts J, Schwartz LH, Sargent D, Ford R, et al. New response evaluation criteria in solid tumours: revised RECIST guideline (version 1.1). Eur J Cancer. 2009;45(2):228-47.
32. Association WM. World Medical Association Declaration of Helsinki: ethical principles for medical research involving human subjects. Jama. 2013;310(
20):2191-4.
