# Supplementary material for: Efficacy of Large Use of Combined Hypofractionated Radiotherapy in a Cohort of Anti-PD-1 Monotherapy-Treated Melanoma Patients
Source: Cancers (Basel). 2022 Aug 23;14(17):4069. doi: 10.3390/cancers14174069 (PMC9454723; doi:10.3390/cancers14174069)
Supplement: Supplementary file 1 [file cancers-14-04069-s001.zip › File S2.pdf]

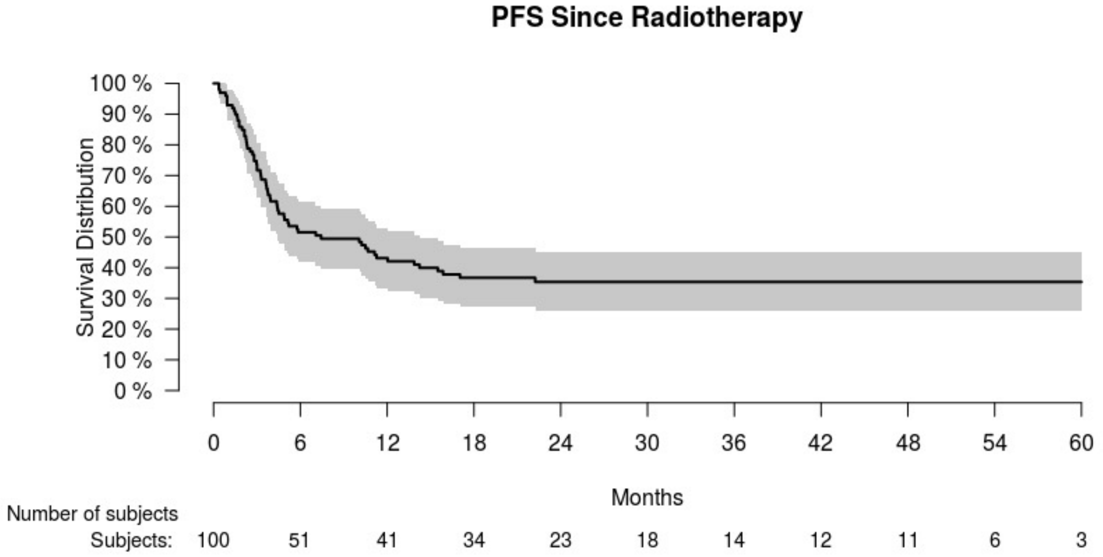

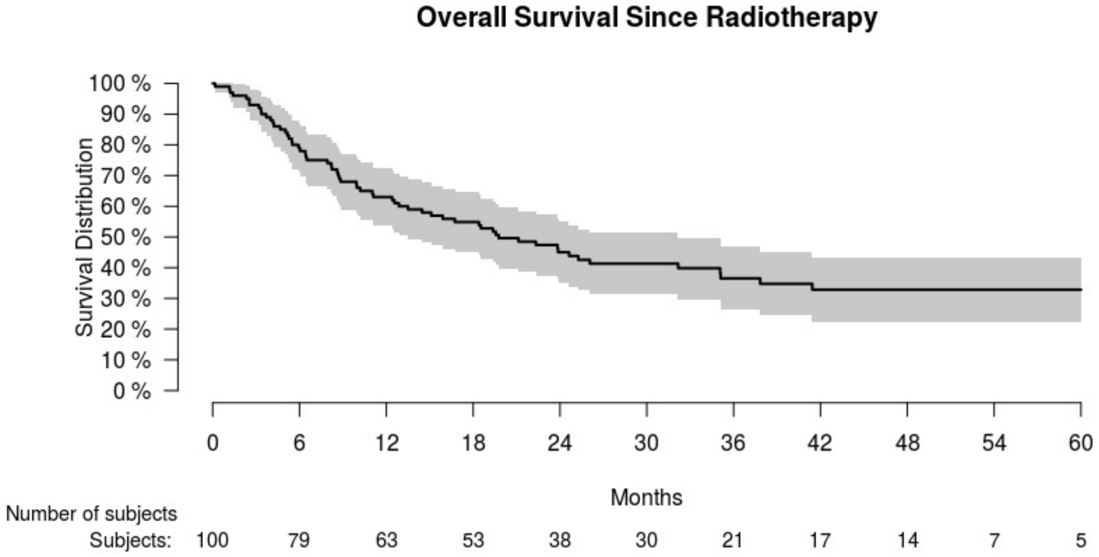

Progression free survival (PFS) and overall survival (OS) in radiated patients from day 1 of radiotherapy
